# Construction of a predictive early warning model based on machine learning neural network for prognosis of patients with traumatic brain injury

**DOI:** 10.3389/fsurg.2026.1741425

**Published:** 2026-03-09

**Authors:** Jun Li, Haoyang Wang, Xiaoli Cao, Lei Sun, Can Zhu, He Li

**Affiliations:** 1 Department of Emergency, The Second Affiliated Hospital of Anhui Medical University, Hefei, Anhui, China; 2 Department of Emergency Medicine, The 901st Hospital of the Joint Logistics Support Force of the People’s Liberation Army of China, Hefei, China

**Keywords:** classification prediction, feature engineering, intelligent algorithm, machine learning, planning model

## Abstract

**Objectives:**

The analysis of prognostic regression of patients in the regional treatment programme for severe trauma can improve the survival rate and quality of life of patients. The aim of this study is to construct an accurate and effective prognostic prediction model for the optimization and development of the regional trauma care network.

**Methods:**

We firstly extracted the clinical data of patients admitted to the regional treatment programme for severe trauma in our hospital during the period from January 2020 to December 2022. The criterion weighting method was adopted to comprehensively evaluated the AIS scores of the cumulative patients in different parts of the body. Based on the regression, the patients were divided into cured group, improved group and poor prognosis group. Based on the dependent variables, the included influencing factors were subjected to univariate analysis, multivariate analysis, and prediction model construction and comparison study. Genetic algorithm was used to solve the planning model; combined with the results of unifactorial analysis and Xgboost, RF was used to screen the features, and the interpretable model (SHAP) and column charts were used to verify the effectiveness of the screened features.

**Results:**

After feature screening and interpretable model validation, 11 indicators such as the main diagnostic score, AIS score and albumin were ultimately included as the important influencing factors of outcome variables, among which albumin was the more important protective factor, and the diagnostic score and AIS score were the more important risk factors. In the comparative study of categorical prediction models, the RF-Transformer-LSTM model achieved the most excellent prediction effect, the accuracy rate of the model test set was 0.9556, the precision rate was 0.9615, the TPR was 0.9474, the TNR was 0.9619, F1 value of 0.9544 as well as AUC value of 0.9271, and in the construction of the three-classification model, the accuracy of the model test set reached 0.9310.

**Conclusion:**

We constructed RF-Transformer-LSTM prediction model has high prediction accuracy and good interpretability in practical applications, which can provide strong support for the optimisation of regional trauma treatment strategies.

## 1 Introduction

The Regional Trauma Care System is a comprehensive medical treatment system that aims to provide timely, standardized, and efficient treatment and care for severely traumatized patients through a regionalized and systematic trauma care network. With the gradual improvement of the trauma emergency system and the promotion of regional care programs, a large number of researchers at home and abroad have begun to pay attention to how to evaluate the prognosis of severely traumatized patients through data analysis and model construction, so as to help formulate more reasonable treatment and intervention strategies (1, 2). Researchers in trauma prediction have gradually developed more refined prognostic prediction models from traditional basic models such as APACHE II score and TRISS score (3, 4). For example, the Injury Severity Score (ISS) is widely used to predict the mortality of trauma patients, and combined with the Revised Trauma Score (RTS), it can conduct preliminary grading of trauma patients (5, 6). How to comprehensively analyze patient information with different injury conditions and improve the interpretability of the model is one of the key research directions at present (7, 8).

In clinical practice, the diagnostic data of trauma patients mostly exist in electronic medical records in the form of free text, including information such as patients’ condition description, trauma type, injury site, and treatment process. Through text information matching and scoring methods, key information in medical records can be extracted and converted into structured data for further analysis and prognostic prediction. Intelligent optimization algorithms can effectively analyze multi-dimensional and complex data, while text recognition can provide more abundant input data for the model. The combination of the two can mine potential risk factors from a wider range of data sources and effectively improve the predictive ability of the model (9, 10).

In recent years, prediction models based on machine learning have become a research hotspot in trauma prognosis. Algorithms such as Random Forest (RF), Support Vector Machine (SVM), Gradient Boosting Tree (XGBoost/LightGBM), and penalized Logistic regression have gradually been introduced into the construction of prognostic prediction models for trauma patients (11, 12). By analyzing multi-dimensional data such as patients’ physiological indicators, laboratory tests, and imaging results, efficient and accurate prognostic prediction can be achieved (13).

This study comprehensively analyzes the information of patients in the regional care program, covering three key links: data extraction, feature screening, and model construction. It provides innovative ideas and methods for the identification and application of clinical text information, influencing factor analysis, and the construction of prognostic prediction models. It not only provides more refined and personalized strategies for trauma treatment, but also opens up a new direction for the optimization and improvement of clinical decision support systems, which has important practical significance and application prospects.

## 2 Methods

**Figure 1:**
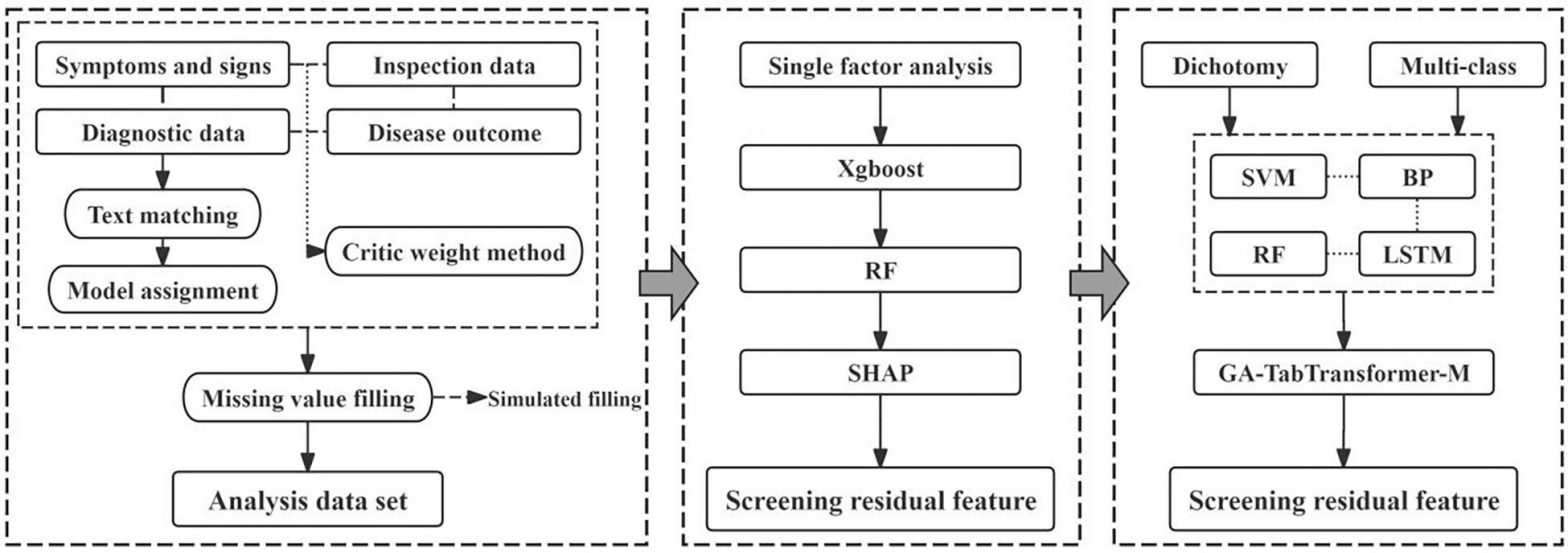
Technical roadmap. M in the flow chart represents the base learner.

This study mainly focuses on three key links: data collection and preprocessing, feature engineering, and model construction and comparison. The overall research framework and method flow are shown in Figure 1.

### 2.1 Research data

The data in this study were obtained from patients who met the criteria of the Regional Severe Trauma Care Program admitted to our hospital from January 2020 to December 2022. The collected data cover four main aspects: symptoms and signs, examination and test results, diagnostic information, and outcome status. Disease outcomes include three outcomes: cure, improvement, and poor prognosis, all of which are clinical outcomes formed by independent evaluation by ≥3 trauma specialists in accordance with uniform operating standards (referring to the 3rd edition of the Clinical Guidelines for Trauma Treatment) and verified for consistency using Cohen's kappa coefficient (*κ* = 0.87). Disputed cases were adjudicated through expert meeting consensus. Finally, a total of 292 patient samples were included (108 cured, 130 improved, and 54 with poor prognosis), including 48 feature variables (1 dependent variable was three-class outcome). The study has been approved by the Ethics Committee [Ethics Review No.: (Ethics No.)]. This study will focus on multi-classification prediction (cure/improvement/poor prognosis) as the main analysis, and plan to compare the efficacy with classic trauma scores such as TRISS and RTS and the penalized Logistic regression baseline model. At the same time, SHAP interpretability analysis will be used to verify the feature contribution.

### 2.2 Inclusion and exclusion criteria

Inclusion criteria: (a). Patient type: All patients who meet the criteria of the Regional Severe Trauma Care Program. That is, they meet the trauma treatment standards formulated by our hospital at the time of admission, and their injury conditions meet the definition of severe trauma (such as traumatic shock, severe traumatic brain injury, extensive burns, etc.). (b). Length of hospital stay: Patients must have complete medical record data records during hospitalization in our hospital, including symptoms and signs, examination and test results, diagnostic information, and outcome status. (c). Data integrity: The relevant data of patients’ clinical symptoms, examination and test results, diagnostic information, and outcome status are relatively complete and meet the research and analysis needs.

Exclusion criteria: (a). Incomplete data: Patients with missing or incomplete key clinical data that cannot meet the research and analysis needs (such as missing symptoms, signs, or examination results). (b) Patients who died in the acute phase: Patients who died immediately after admission or died of irreversible failure caused by trauma in a short period of time, and effective outcome analysis cannot be performed. (c) Non-traumatic diseases: Patients admitted due to non-traumatic diseases (such as stroke, heart disease, etc.) that do not meet the severe trauma criteria. (d) Duplicate records: For patients with duplicate hospitalization records during the study period, only their first hospitalization data were selected for analysis.

### 2.3 Text matching and comprehensive scoring

#### 2.3.1 Extraction of diagnostic information

Structured extraction of patients’ main diagnostic information and other diagnostic information was carried out: the main diagnosis was divided into 7 modules: head and cranium, neck, chest, abdomen, fracture (limb/pelvis), spine and spinal cord, and multiple injuries; other diagnoses were divided into 7 modules: hemorrhagic, organ injury, cardiovascular, soft tissue, fracture, vertebra, and underlying diseases. Each module is equipped with an exclusive keyword text library and weight coefficient. The specific score F of each patient's diagnostic information is obtained by accumulating the product of the number of keyword matches in each module and the weight coefficient. The calculation process can be expressed as (Equation 1):

$$F_{i}=\sum_{j=1}^{7}\delta_{\mathrm{ij}}N_{\mathrm{ij}}$$ (1)

Among them, represents the patient number, represents the diagnostic module, represents the module weight coefficient, and represents the number of keyword matches in the patient's diagnostic information for that module. The weight coefficient was determined by constructing a planning model: based on the principle of information entropy, the fluctuation degree of diagnostic text sequence data is positively correlated with its information carrying capacity: the greater the data fluctuation, the more significant the uncertainty characteristic of information entropy, corresponding to higher effective information density. To ensure that the extracted text features have clinical discriminative value, this study takes the overall variance of the diagnostic text sequence data as the objective function, and enhances the data dispersion by maximizing the variance quantitative index, so that the generated feature data can better reflect the diagnostic differences among different patient groups, and improve the representativeness and accuracy of subsequent model construction (Equation 2).

$$\max\sigma^{2}=\frac{1}{n}\overset{n}{\underset{i\;=\;1}{\sum }}\;{\left(F_{i}\text{-}\overset{-}{F}\right)}^{2}$$ (2)

**Figure 2:**
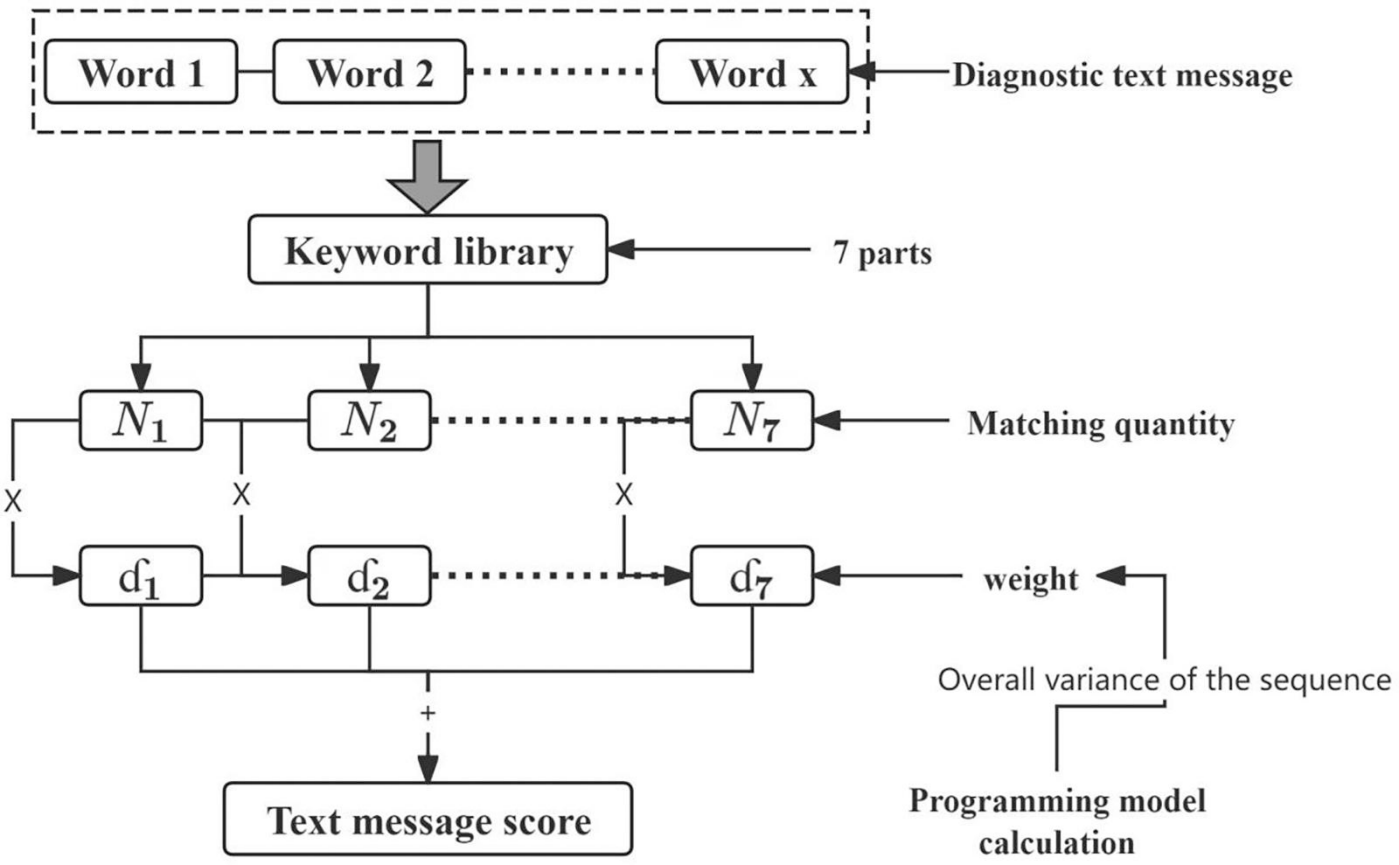
Schematic diagram of text matching and scoring process.

Taking the weight of each diagnostic module as the decision variable, to avoid extreme values in weight allocation, the weight range is limited to [0,5]. The Genetic Algorithm was used to solve the optimal weight configuration. This algorithm simulates the selection, crossover, and mutation mechanisms in the biological evolution process, and can efficiently search for the global optimal solution in the multi-dimensional weight space. It is especially suitable for the complex optimization scenario of multi-module weight coupling and nonlinear objective function in this study. It can effectively avoid the problem that traditional gradient descent methods are easy to fall into local optimality, and can flexibly handle weight range constraints, and finally realize the global optimal configuration of diagnostic module weights (Figure 2).

#### 2.3.2 Comprehensive evaluation of AIS score

The AIS score mainly scores injuries to different organs and tissues of patients, and is often used in trauma research, clinical evaluation, and trauma prediction models (14, 15). This study comprehensively analyzes patients’ information of different types and parts. To realize the comparability of AIS scores, it is necessary to comprehensively evaluate the AIS scores of various systems and parts.

The CRITIC weighting method is a method used in Multi-Attribute Decision Making (MADM), which calculates the weight of each feature by evaluating the correlation and information content between different features. In the outcome analysis of trauma patients, the CRITIC method can comprehensively consider the impact of AIS scores of various parts on patients’ prognosis, so as to obtain a more reasonable weight allocation and comprehensive evaluation results. In addition, there may be a certain degree of information redundancy in various parts of the AIS score (for example, chest trauma and abdominal trauma may jointly affect the patient's respiratory function). The CRITIC method can identify which features are highly correlated and make appropriate weight adjustments to them, thereby reducing the impact of redundant information and avoiding a certain feature overdominating the comprehensive evaluation results (16, 17). This study positions the CRITIC weighting method as a data-driven exploratory comprehensive indicator, which does not replace the existing ISS/NISS standard scoring system. In the main analysis, ISS/NISS also enters the screening model as an inclusion standard indicator.

To make full use of patients’ AIS score information and realize accurate prediction of disease outcomes, combined with expert consensus and previous studies, AIS scores of 5 parts were included for comprehensive evaluation: head and neck (including cervical spine), face (including facial cranium), chest (including thoracic spine and diaphragm), abdomen and pelvis (including lumbar spine), and limbs and pelvis.

To verify the stability of the method, this study set up three parallel weighting schemes for comparison: (a). Equal-weight average comprehensive AIS; (b). ISS/NISS standard method; (c). CRITIC comprehensive weight. The evaluation through discriminative efficiency, calibration degree, and Decision Curve Analysis (DCA) shows that the core conclusions (the relative importance of head and neck and chest AIS scores) are consistent under different weighting schemes; if the CRITIC scheme does not show statistical or clinical advantages, the conclusion will prioritize recommending ISS/NISS or the equal-weight scheme as the clinical deployment option.

### 2.4 Statistical analysis methods

#### 2.4.1 Univariate analysis

According to the dependent variable divided into 3 categories, measurement data were expressed as. Analysis of variance or Kruskal–Wallis *H* test was used according to the results of normality test. Count data were expressed as the number of cases, and chi-square test was used for inter-group comparison.

#### 2.4.2 Multivariate analysis

Xgboost, RF, and SHAP interpretable models were used for feature screening (RF was used for feature importance ranking and robustness screening), and the final scale of variables entering the model was controlled according to the event number-variable number constraint (EPV principle). Multivariate analysis methods were used to evaluate the independent impact of each variable on patients’ prognostic outcomes during hospitalization (18, 19). Logistic regression analysis was used to estimate the independent impact of each variable on prognostic outcomes, and the odds ratio (OR) of each variable was calculated to evaluate its importance in prognosis and draw a nomogram.

#### 2.4.3 Construction of prediction model

Based on the results of univariate analysis and multivariate analysis, binary and multi-class prognostic prediction models were constructed according to the classification of dependent variables to provide support for clinical decision-making. The main analysis models adopted XGBoost, LightGBM, and penalized Logistic regression (L1/L2 regularization), and TabTransformer was used as the sensitivity analysis model (for high-cardinality categorical feature embedding). The Genetic Algorithm (GA) was used to optimize the feature subset and hyperparameters, named “GA-TabTransformer + classifier” (20).

#### 2.4.4 Model evaluation and verification

The division ratio of the training set and test set was 8:2. A nested cross-validation strategy was adopted (outer layer 5-fold stratified validation, inner layer combined with Genetic Algorithm for hyperparameter tuning), and an internal-external verification mechanism of time division was introduced (the latest time window data was set as an independent time external verification set). The evaluation indicators adopted a multi-dimensional system, including Accuracy, Sensitivity, Specificity, F1 score, ROC Curve (Receiver Operating Characteristic Curve), and AUC value (Area Under Curve), and 95% confidence interval and calibration performance evaluation were calculated. At the same time, model complexity and benefit evaluation (parameter scale, training/inference time) and ablation experiments were carried out to verify the rationality of feature screening and model architecture (21, 22).

#### 2.4.5 Statistical software and hypothesis testing

Data analysis was processed using SPSS 24.0, R 4.4.2, and MATLAB 2024a statistical analysis software. The significance level of hypothesis testing was set at 0.05. If the *p*-value was less than 0.05, the factor was considered statistically significant.

#### 2.4.6 Missing value handling

Simulated imputation was used for missing value imputation, including mean imputation, mode imputation, median imputation, and multiple imputation. According to the changes of the imputation sequence, the missing value imputation method with the smallest degree of change was finally selected (23).

## 3 Results

The results section mainly includes the data preprocessing process, statistical analysis results, and comprehensive evaluation of model prediction efficiency, specifically the key links of data cleaning and feature engineering of included data, significance analysis of various influencing factors, and classification performance verification of prediction models.

### 3.1 Text matching and comprehensive evaluation

#### 3.1.1 Calculation of text matching weights

According to the clinical diagnostic characteristics of medical records, diagnostic information was divided into main diagnostic information and other diagnostic information, and quantitative processing was carried out by combining text structured matching with multi-dimensional weight assignment: first, diagnostic keywords were extracted through natural language processing technology, and then a three-dimensional weight calculation model of “diagnostic type-injury site-keyword importance” was constructed by combining trauma severity classification standards and clinical prognosis correlation analysis, and finally an individualized diagnostic score for each patient was generated. The weight assignment logic was mainly based on: (a). The decisive impact of the main diagnosis on prognosis (such as multiple injuries, craniocerebral injuries); (b). The interference degree of complications on treatment decisions (such as hemorrhagic diseases, cardiovascular injuries); (c). The anatomical importance of the trauma site (such as spinal cord injury, abdominal organ rupture). The weight calculation results are shown in Table 1, and the text information of diagnostic keywords is shown in Supplementary Table 1 and the GA convergence curve is shown in Supplementary Figure 2 in Supplementary Materials.

**Table 1:** Summary of weight calculation results.

Category	Module	Keyword	Weight	Objective function
Primary diagnosis	Head and brain	Concussion, craniocerebral injury, etc	5	4.008
	Neck	Cervical spine injury, cervical spine fracture, etc	1	
	Thorax	Rib fracture, lung contusion, etc	2	
	Belly	Abdominal hemorrhage, splenic rupture, etc	3	
	Fracture (limb/pelvis)	Fracture, fracture reduction, etc	1	
	Spine and spinal cord	Spinal fracture, spinal cord injury, etc	3	
	Multiple injuries	Multiple trauma, multi-site injury, etc	5	
Other diagnosis	Hemorrhagic	Hematoma, internal bleeding, etc	5	42.005
	Organ injury	Liver injury, kidney injury, etc	1	
	Cardiovascular	Heart injury, pericardial effusion, etc	5	
	Soft tissue	Muscle injury, ligament tear, etc	1	
	Fracture	Open fracture, closed fracture, etc	1	
	Cone	Spinal cord injury, nerve injury, etc	1	
	Underlying disease	Diabetes, high blood pressure, etc	4	

The results in Table 1 show that the weight distribution presents significant clinical priority characteristics: in the main diagnosis category, craniocerebral injury (weight = 5) and multiple injuries (weight = 5) obtained the highest scores, which is consistent with the clinical consensus in trauma treatment that “craniocerebral injury has a high mortality rate and multiple injuries have a high risk of complications”. Its objective function value of 4.008 indicates that the sequence value calculated according to this weight has the largest fluctuation, and the overall variance value is 4.008; spinal cord injury (weight = 3) and abdominal injury (weight = 3) are the next, reflecting the key impact of thoracoabdominal organ injury and spinal cord dysfunction on treatment decisions. In the other diagnosis category, hemorrhagic diseases (weight = 5) and cardiovascular injuries (weight = 5) were also assigned high weights, which are directly related to the high mortality risk of coagulation dysfunction and heart failure after trauma. Underlying diseases (weight = 4), although lower than acute trauma, are still higher than non-fatal injuries such as soft tissue injuries (weight = 1), suggesting the potential interference of chronic diseases on the trauma repair process. The above weight distribution rules provide a clinical anchor for subsequent feature screening, ensuring that the model can extract the value of diagnostic information to the greatest extent.

#### 3.1.2 Comprehensive evaluation

To comprehensively use patients’ injury information, it is necessary to comprehensively evaluate the AIS scores of patients’ different parts. This study set up three parallel weighting schemes for comparison: (a). Equal-weight average comprehensive AIS; (b). ISS (Injury Severity Score) standard method; (c). CRITIC objective weight method. The comprehensive evaluation results and performance comparison of the three schemes are shown in Table 2. A comprehensive score for each patient was calculated according to the weight assignment results and inter-group comparison was carried out. The dependent variable was set as a binary variable according to clinical outcomes (cure/improvement combined as good prognosis group, others as poor prognosis group), and multi-classification analysis was carried out simultaneously in the prediction stage.

**Table 2:** Comparison of comprehensive evaluation results of three weighting schemes.

Weighting scheme	Head and neck weight (%)	Face (%)	Chest (%)	Limbs and pelvis (%)	Abdomen and pelvis (%)	Surface of the body (%)	Discrimination (AUC)	Calibration (H-L p)
Equal weight	0.167	0.167	0.167	0.167	0.167	0.167	0.394	0.199
ISS standard	0.471	0.055	0.199	0.100	0.128	0.047	0.723	0.339
CRITIC weight	0.483	0.065	0.204	0.102	0.103	0.043	0.718	0.100

The ISS method itself is not a weighted sum, but directly calculates the ISS score (the sum of the squares of the top 3 highest scores). The “weight” in the table is only used for result examples, indicating the average contribution of each part in the ISS calculation.

According to the data analysis in Table 3, all three weighting schemes show that the AIS scores of the head and neck and chest have a significantly higher predictive effect on patients’ outcomes than other parts (CRITIC weights are 48.3% and 20.4%, respectively; ISS standards are 47.1% and 19.9%, respectively). There are gradient differences in the discriminative efficiency of different schemes: the ISS standard (AUC = 0.723) is close to the CRITIC weight method (AUC = 0.718), which is significantly better than the equal-weight average scheme (AUC = 0.394); in terms of calibration degree, both the CRITIC weight method (H-L *p* = 0.100) and the ISS standard (H-L *p* = 0.339) meet the statistical requirements (*p* > 0.05). The core conclusion (the relative importance of the head and neck and chest) is consistent under the three weighting schemes. Among them, the CRITIC weight method dynamically optimizes the weight allocation through index variability (such as 1.387 for the chest) and conflict (such as 0.660 for the head and neck), which is more in line with the data characteristics than the ISS standard. This study positions the CRITIC weight method as a data-driven exploratory comprehensive indicator, which does not replace the ISS standard system, and is included in the model as an independent variable for analysis; in clinical deployment, if the CRITIC scheme does not show statistical or clinical advantages, the ISS standard scheme is preferred.

**Table 3:** Results of weight calculation by CRITIC weighting method.

Item	Index variability	Index conflict	Amount of information	Weight (%)
Head and neck (including cervical spine)	1.176	0.660	0.776	48.3
Face (including facial cranium)	1.031	0.729	0.752	6.5
Chest (including thoracic vertebra and diaphragm)	1.387	0669	0.928	20.4
Limbs and pelvis	1.486	0.734	1.091	10.2
Abdomen and pelvis (including lumbar spine)	1.475	0.617	0.910	10.3
Surface of the body	0.560	0.745	0.417	4.3

### 3.2 Results of univariate analysis

After processing the text information of medical records and comprehensive evaluation of AIS scores, this study finally determined 48 variables for analysis, including 292 patients, among which 108 were cured, 130 were improved, and 54 had poor prognosis. Some variables had missing data, but the proportion of missing values was all less than 5%, which met the inclusion criteria. For missing values, simulated imputation was used for processing. After comparing various imputation methods, the results showed that mode imputation had the least impact on the changes of the original data sequence. Therefore, this study used mode imputation to handle missing values, and performed univariate analysis on the imputed data to further explore the relationship between each variable and patient outcomes. The results of univariate analysis are shown in Table 4.

**Table 4:** Results of univariate analysis.

Trait	Group			Statistic	*p*-value
	Cure group (108)	Improved group (130)	Poor prognosis group (54)		
Age	54.5 (46.0, 65.0)	55.0 (41.0, 67.0)	58.5 (47.0, 73.0)	4.666	0.097
Major diagnostic scores	3.0 (2.0, 5.0)	5.0 (5.0, 5.0)	5.0 (5.0, 5.0)	71.384	<0.001
Other diagnostic scores	5.0 (3.0, 10.0)	8.0 (4.0, 13.0)	14.5 (9.0, 18.0)	35.523	<0.001
AIS score(Weighted)	1.3 (1.0, 1.6)	1.6 (1.3, 2.0)	1.7 (1.4, 2.1)	29.254	<0.001
ISS score	20.0 (17.0, 25.0)	22.0 (18.0, 29.0)	29.0 (23.0, 35.0)	36.526	<0.001
temperature (℃)	36.5 (36.4, 36.8)	36.7 (36.5, 36.9)	36.6 (36.5, 36.8)	5.063	0.080
Heart rate (times/min)	85.0 (75.5, 90.0)	87.5 (75.0, 102.0)	100.0 (78.0, 119.0)	14.577	0.001
Breathing (cycles per minute)	19.0 (18.0, 20.0)	20.0 (17.0, 22.0)	19.0 (16.0, 24.0)	0.831	0.660
Systolic pressure (mmHg)	127.5 (115.5, 144.5)	127.5 (112.0, 144.0)	125.0 (98.0, 144.0)	1.152	0.562
Diastolic blood pressure (mmHg)	80.0 (71.5, 89.0)	75.0 (65.0, 84.0)	72.0 (57.0, 85.0)	10.962	0.004
HB(g/L)	121.5 (108.0, 136.0)	123.0 (102.0, 135.0)	103.0 (81.0, 124.0)	17.141	<0.001
Prothrombin time	18.5 (7.0, 36.5)	22.0 (11.0, 45.0)	24.0 (10.0, 59.0)	4.572	0.102
International normalized ratio	21.0 (8.5, 38.5)	22.0 (8.0, 35.0)	22.5 (9.0, 52.0)	1.230	0.541
Activated partial prothrombin time	49.5 (33.0, 84.0)	54.5 (25.0, 84.0)	53.5 (19.0, 105.0)	0.722	0.697
Thrombin time	28.0 (13.5, 45.0)	29.5 (12.0, 49.0)	23.0 (10.0, 58.0)	0.121	0.941
Fibrinogen	86.0 (46.0, 123.5)	72.0 (38.0, 127.0)	81.0 (29.0, 113.0)	1.710	0.425
Total bilirubin	14.4 (9.8, 20.0)	13.4 (9.4, 18.1)	11.9 (8.5, 16.1)	3.673	0.159
Direct bilirubin	5.7 (3.9, 8.2)	5.2 (3.8,6 .8)	5.3 (3.4, 6.7)	4.775	0.092
Indirect bilirubin	8.1 (6.0, 12.0)	7.9 (6.1, 11.1)	7.2 (5.8, 9.2)	2.877	0.237
Total protein	59.8 (55.2, 64.8)	59.7 (52.9, 65.3)	56.6 (47.5, 63.0)	7.577	0.023
Albumin	38.1 (35.0, 41.0)	38.0 (35.1, 41.8)	34.8 (29.3, 40.0)	10.761	0.005
Globulin	21.6 (18.2, 24.8)	21.3 (18.1, 24.3)	19.5 (16.7, 23.5)	3.244	0.197
White-sphere ratio	1.8 (1.5, 2.1)	1.8 (1.6, 2.1)	1.7 (1.4, 2.0)	4.125	0.127
Glutamic-pyruvic transaminase	27.3 (18.3, 52.4)	26.1 (16.3, 56.6)	30.1 (18.4, 65.5)	1.145	0.564
Glutamic oxalacetic transaminase	32.1 (21.9, 56.0)	34.4 (25.1, 61.3)	51.0 (31.5, 90.5)	10.486	0.005
Millet grass/millet C	1.3 (1.0, 1.6)	1.4 (1.1, 1.8)	1.2 (1.5, 2.1)	7.056	0.029
Alkaline phosphatase	62.0 (50.5, 78.0)	61.5 (50.3, 80.0)	63.5 (46.0, 81.0)	0.382	0.826
Total bile acids	2.7 (1.3, 5.4)	2.4 (1.1, 4.6)	2.4 (0.8, 4.4)	0.553	0.758
prealbumin	250.0 (203.0, 294.0)	262.5 (204.0, 294.0)	226.0 (134.0, 263.0)	6.282	0.043
*α*-L fucosidase	21.8 (18.3, 25.9)	22.8 (18.6, 25.7)	25.7 (17.5, 25.7)	0.776	0.678
Adenosine deaminase	7.0 (5.0, 10.4)	7.0 (5.0, 10.0)	5.0 (5.0, 7.6)	6.132	0.047
Cholinesterase	6,162 (4,973, 7,489)	5,948 (4,569, 7,559)	5,305 (4,569, 6, 507)	6.528	0.038
Mitochondrial aspartate transferase	15.4 (9.7, 24.3)	14.3 (9.8, 24.3)	16.7 (9.5, 26.6)	1.575	0.455
Urea	6.0 (4.8, 7.3)	5.6 (4.5, 7.1)	6.2 (4.9, 8.0)	3.274	0.195
Creatinine	65.0 (57.1, 77.5)	59.9 (50.0, 78.3)	75.7 (54.3, 104.3)	10.694	0.005
Number of transfusion	0.0 (0.0, 1.0)	0.0 (0.0, 2.0)	0.0 (1.0, 2.0)	5.120	0.077
Rescue times	0.0 (0.0, 0.0)	0.0 (0.0, 0.0)	0.0 (1.0, 2.0)	49.662	<0.001
Headache (No: Yes: coma)	49:49:10	29:68:33	4:18:32	59.304	<0.001
Vomiting (No: Yes)	88:20	79:51	28:26	18.064	<0.001
Meningeal irritation (No: Yes)	104:4	120:10	51:3	1.720	0.423
Open airway (No: Yes)	97:11	97:33	19:35	54.771	<0.001
Cranial pressure reduction therapy (No: yes)	106:2	122:8	47:7	8.153	0.017
Prevent infection (No: Yes)	90:18	108:22	50:4	3.041	0.219
Antishock therapy (No: yes)	91:17	106:24	40:14	2.464	0.292
Gender (female: Male)	21:87	44:86	15:39	6.156	0.046
Type of injury (Traffic: Fall: Other)	47:40:21	67:51:12	26:22:6	5.794	0.215
Surgery (No: Yes)	22:86	42:88	26:28	13.270	0.001

Count data in the table are expressed as the number of cases, and chi-square test is used for inter-group comparison. After normality test, measurement data do not conform to normal distribution, so they are expressed as M (P25, P75), and Kruskal–Wallis Test is used for inter-group comparison.

According to the results of univariate analysis, 21 indicators including main diagnosis score, other diagnosis score, comprehensive AIS score, ISS score, heart rate, diastolic blood pressure, hemoglobin (HB), total protein, albumin, aspartate aminotransferase, aspartate/alanine ratio, prealbumin, adenosine deaminase, cholinesterase, creatinine, number of rescues, headache symptoms, vomiting symptoms, whether the airway is open, gender, and whether surgery is performed showed significant statistical differences among different groups. For specific differences, refer to the median (M) and interquartile range (P25, P75) of indicators in each group.

### 3.3 Feature engineering

In the univariate analysis, a total of 21 indicators showed significant statistical differences among different groups (*P* < 0.05). Considering that the two indicators of the number of rescues and whether surgery was performed failed to be completely collected at the time of admission in some hospitalized cases, with timeliness and completeness limitations, they were excluded before model construction. The remaining 19 indicators were included in the subsequent feature screening process.

#### 3.3.1 Feature screening

The indicators screened by univariate analysis may have collinearity problems, leading to feature redundancy. Therefore, reasonable feature screening is needed to optimize the model input. In the feature engineering stage, based on the core goal of clinical prognostic evaluation and referring to the AIS score weight fusion logic, cured and improved cases were combined into a “good prognosis group”, which formed a binary outcome variable with the poor prognosis group (238 cases in the good prognosis group and 54 cases in the poor prognosis group). The sample data were significantly imbalanced (about 4.4:1). To reduce its impact on model training, this study adopted various sampling strategies for data preprocessing: Random Forest (RF) was used as the base learner, the training set and test set were divided at 8:2, and the model performance of different sampling methods was compared through 5-fold cross-validation (with AUC and F1 value as the main evaluation indicators). The results showed that the SMOTE-ENN (Synthetic Minority Oversampling Technique combined with Edited Nearest Neighbors undersampling) combined sampling method had the best performance. After processing, the sample ratio of the two groups was optimized to 1:1.4 (93:130). RF and XGBoost algorithms were used for feature importance ranking, with a feature importance threshold of 5% as the screening standard (Table 5). After modeling different data sets with LightGBM respectively, it was found that the intersection test set result of the two algorithms’ screening results was the best, and its test set AUC value reached 0.89, which was much better than other data sets (Table 6). A total of 11 core features were retained, including: main diagnosis score, other diagnosis score, CRITIC weight method AIS comprehensive score, heart rate, hemoglobin (Hb), albumin, aspartate aminotransferase, prealbumin, adenosine deaminase, cholinesterase, and creatinine.

**Table 5:** Characteristic screening results.

Method	Quantity	Retention feature
RF	10	Glutamic oxalacetic transaminase, cholinesterase, AIS score (weight), albumin, other diagnostic score, Primary diagnostic score, HB, creatinine, prealbumin, adenosine deaminase
Xgboost	8	Albumin, adenosine deaminase, glutamic oxalacetic transaminase, HB, other diagnostic scores, prealbumin, heart rate, AIS score (weight)

**Table 6:** Model evaluation metrics of Various datasets (LightGBM).

Dataset	Characteristic number	Data set	ACC	PRE	TPR	TNR	F1	AUC
D1	10	Training set	0.941	0.930	0.972	0.899	0.951	0.993
		Test set	0.761	0.767	0.852	0.632	0.807	0.848
D2	8	Training set	0.941	0.938	0.963	0.911	0.950	0.989
		Test set	0.783	0.774	0.889	0.632	0.828	0.833
D3	7	Training set	0.915	0.927	0.927	0.899	0.927	0.972
		Test set	0.739	0.759	0.815	0.632	0.786	0.779
D4	5	Training set	0.952	0.955	0.963	0.937	0.959	0.996
		Test set	0.826	0.828	0.889	0.737	0.857	0.890

Note: D1 represents the RF-screened feature set; D2 represents the Xgboost-screened feature set; D3 represents the union of D1 and D2; D4 represents the intersection of D1 and D2.

#### 3.3.2 SHAP interpretable model

**Figure 3:**
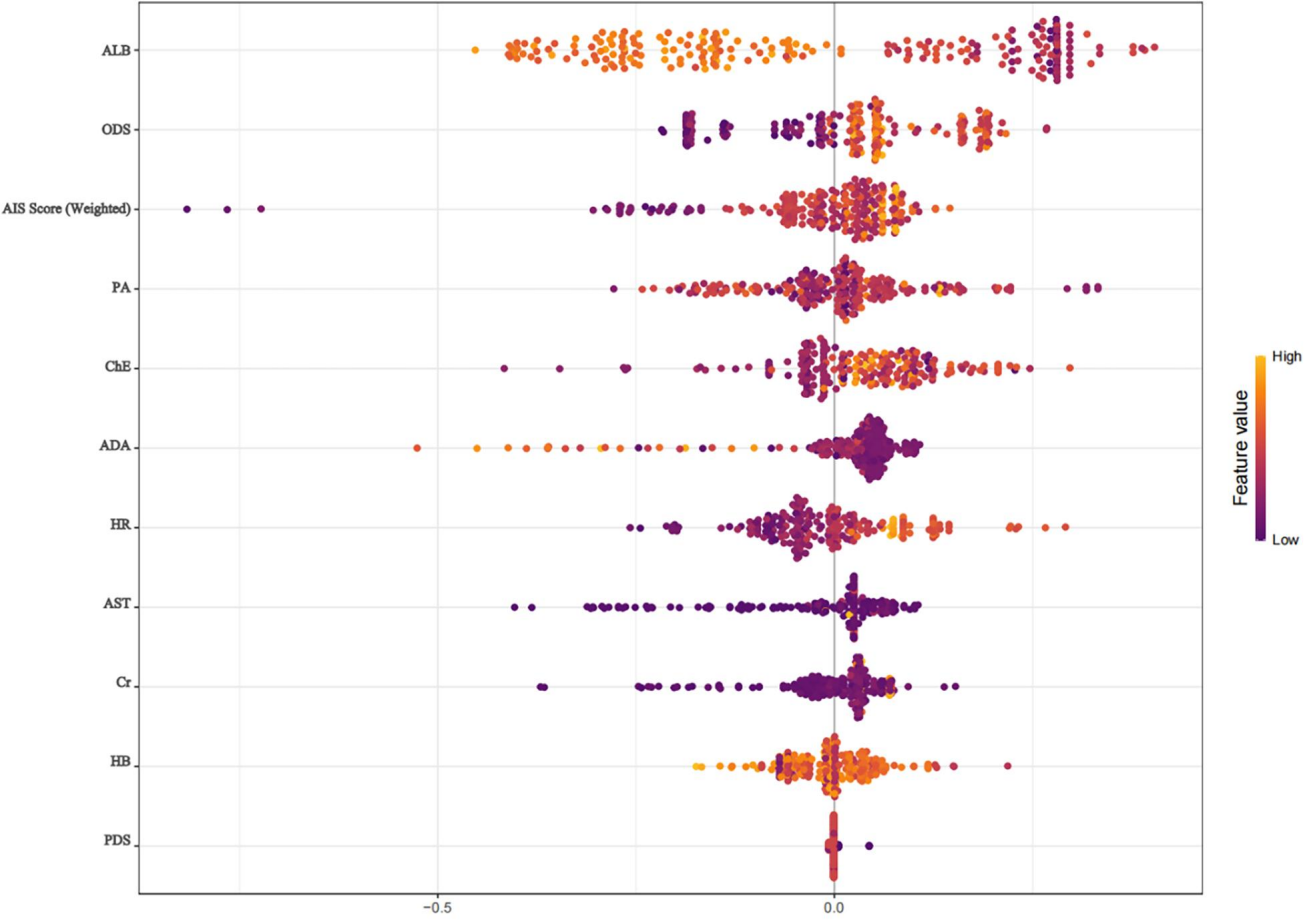
SHAP swarm diagram.

**Figure 4:**
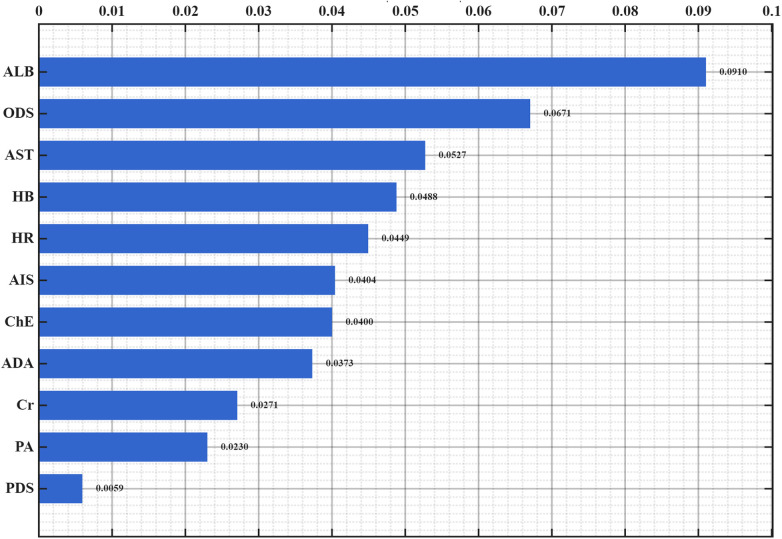
Ranking of feature importance.

The SHAP interpretable model was used for quantitative analysis of the included 11 variables (Figure 3). The importance ranking was carried out by calculating the mean absolute SHAP value of the features, and the positive and negative distribution of SHAP values was combined to reveal the impact direction of each indicator on prognosis (Figure 4). The results showed that the specific SHAP values of each included indicator were: albumin (ALB, 0.0910), other diagnosis score (ODS, 0.0671), aspartate aminotransferase (AST, 0.0527), hemoglobin (HB, 0.0488), heart rate (HR, 0.0449), comprehensive AIS score (0.0404), cholinesterase (ChE, 0.0400), adenosine deaminase (ADA, 0.0373), creatinine (Cr, 0.0271), prealbumin (PA, 0.0230), main diagnosis score (PDS, 0.0059).

Factors increasing the risk of poor prognosis include: ALB (55.61% positive SHAP ratio, high value indicates increased risk), HR (43.50%), ChE (60.99%), ADA (72.20%), PA (56.05).

The top three features (ALB, ODS, AST) cumulatively contributed 42.3% of the model prediction variation. Among them, ALB, as the strongest influencing factor (SHAP value 0.0424 ± 0.1020), its decreased level was significantly correlated with poor prognosis; the negative correlation between ODS and AIS score (average SHAP values −0.0236 and −0.0208) indicated the correction value of the diagnostic scoring system for prognostic evaluation. The SHAP value distribution of all features showed that the model decision path had clinical interpretability, providing a quantitative basis for the screening of biomarkers for trauma prognosis.

#### 3.3.3 Multivariate analysis

To verify the robustness of the previous feature screening results, this study used a binary Logistic stepwise regression model (backward method, α entry = 0.05, *α* exit = 0.10) for multivariate analysis. Variables were screened through likelihood ratio test and AIC value minimization criterion, and the results were visualized with a nomogram. The stepwise regression iteration process showed (Table 7) that the model achieved the optimal fit in the 5th step (AIC = 189.6), and finally included 5 independent influencing factors: other diagnosis score (ODS), comprehensive AIS score (AIS Score, Weighted), and heart rate (HR) were risk factors (*P* < 0.001), and albumin (ALB) and adenosine deaminase (ADA) were protective factors (*P* < 0.01). Among them, the comprehensive AIS score had the strongest impact [Exp(B) = 6.736], indicating the decisive role of trauma severity on prognosis. Model verification results showed: test set AUC = 0.733 (95%CI:0.552–0.914), calibration curve Hosmer-Lemeshow test *P* = 0.342, indicating that the variable combination had good discriminative ability and calibration degree, further confirming the effectiveness of previous feature screening.

**Table 7:** Results of logistic stepwise regression analysis.

Variables in the Equation		B	S.E.	Wald	Sig.	Exp(B)	95% C.I.for EXP(B)	
							Lower	Upper
Step 5^e^	ODS	0.142	0.036	15.147	0.000	1.153	1.073	1.238
	AIS Score (Weighted)	1.907	0.545	12.236	0.000	6.736	2.313	19.612
	HR	0.045	0.012	15.469	0.000	1.046	1.023	1.070
	ALB	−0.092	0.032	8.470	0.004	0.912	0.857	0.970
	ADA	−0.207	0.067	9.667	0.002	0.813	0.713	0.926
	Constant	−3.762	1.997	3.547	0.060	0.023		

**Figure 5:**
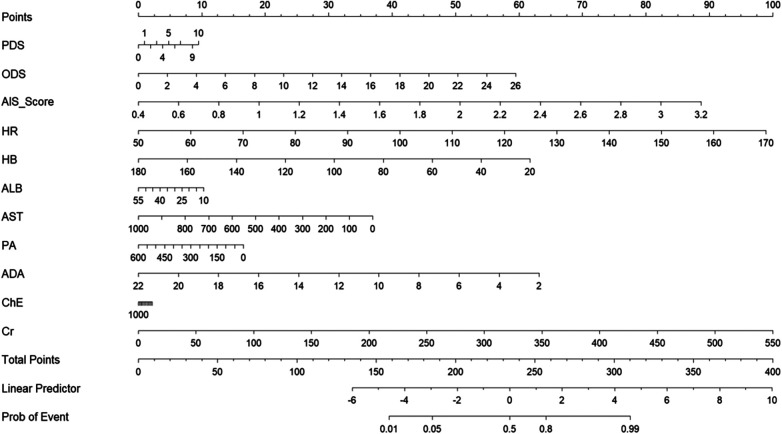
Logisctic regression column line graph. The AIS score value in the figure is the comprehensive value after weight calculation.

A nomogram model was constructed based on the results of multivariate regression (Figure 5). The contribution weight of each variable to prognosis was quantified through visualization: comprehensive AIS score (weight coefficient 0.32), ODS (0.28), and HR (0.21) were the main contributing factors, and ALB (0.12) and ADA (0.07) were secondary protective factors. Internal verification of the nomogram showed: C-index = 0.726 (95%CI: 0.681–0.771), and the calibration curve of 1,000 bootstrap samples fitted well with the ideal curve (mean absolute error = 0.042), indicating that the model had good clinical applicability, and the patient's prognostic risk could be quickly estimated through the sum of variable integrals.

### 3.4 Model construction research

To fully verify the effectiveness of the included variables and construct a clinically applicable prognostic prediction model, this study adopted a three-stage research framework of “feature screening-model comparison-clinical control”: first, RF and XGBoost were combined for feature importance ranking and stability screening (eliminating highly collinear features), and 11 core variables were retained; then gradient boosting tree (XGBoost) and penalized Logistic regression were used as the main analysis models, and TabTransformer for tabular data was used as sensitivity analysis (to explore feature column interactions). The study will simultaneously carry out the construction of binary classification (good/poor prognosis) and multi-classification (cure/improvement/poor prognosis) models, and conduct inter-model comparison research.

For the binary classification problem, the SMOTE-ENN combined sampling method was used to handle data imbalance (the sample ratio after processing was 1:1.4); the multi-classification model was trained with the original data set. Data division adopted a nested cross-validation strategy: the outer layer was 5-fold stratified sampling (training set: test set = 8:2), and the inner layer optimized hyperparameters through Genetic Algorithm (GA); at the same time, time division verification was introduced (the latest time window data was set as an independent verification set). Model evaluation adopted a multi-dimensional indicator system: binary classification tasks reported AUC, F1 value, sensitivity/specificity, and calibration curve; multi-classification tasks added macro/weighted F1, confusion matrix, and class-specific analysis. If complex models do not show statistical or clinical advantages, the simple scheme of XGBoost + nomogram is preferred for clinical deployment to ensure model interpretability and generalization ability.

#### 3.4.1 Binary classification prediction modeling

To systematically evaluate the predictive efficiency of different algorithms on the balanced data set, this study constructed the following model system: benchmark models include penalized Logistic regression (L1 regularization), Support Vector Machine (SVM), Random Forest (RF), and Gradient Boosting Tree (XGBoost); at the same time, TabTransformer for tabular data was used as a sensitivity analysis model to construct combined models with RF and XGBoost respectively, and Genetic Algorithm was used for parameter optimization.

Penalized Logistic regression: L1 regularization (Lasso) was used to control the number of features, the regularization strength (C) was optimized through 5-fold cross-validation, and solver selected liblinear to adapt to small sample scenarios.

Support Vector Machine (SVM): The kernel function adopted Radial Basis Function (RBF) to capture nonlinear relationships. The penalty parameter (C = 1.0) and kernel coefficient (*γ* = 0.1) were determined through grid search; class weight (class_weight = ‘balanced’) was used to balance the sample distribution, and tolerance (tol = 1e-3) controlled the convergence accuracy.

Gradient Boosting Tree (XGBoost): The tree structure parameters were set to max_depth = 6 and min_child_weight = 3 to prevent overfitting; the learning rate (learning_rate = 0.05) and number of iterations (n_estimators = 200) were optimized through GA; gamma = 0.1 was used to control the leaf node splitting threshold, and subsample = 0.8 to enhance generalization ability.

**Figure 6:**
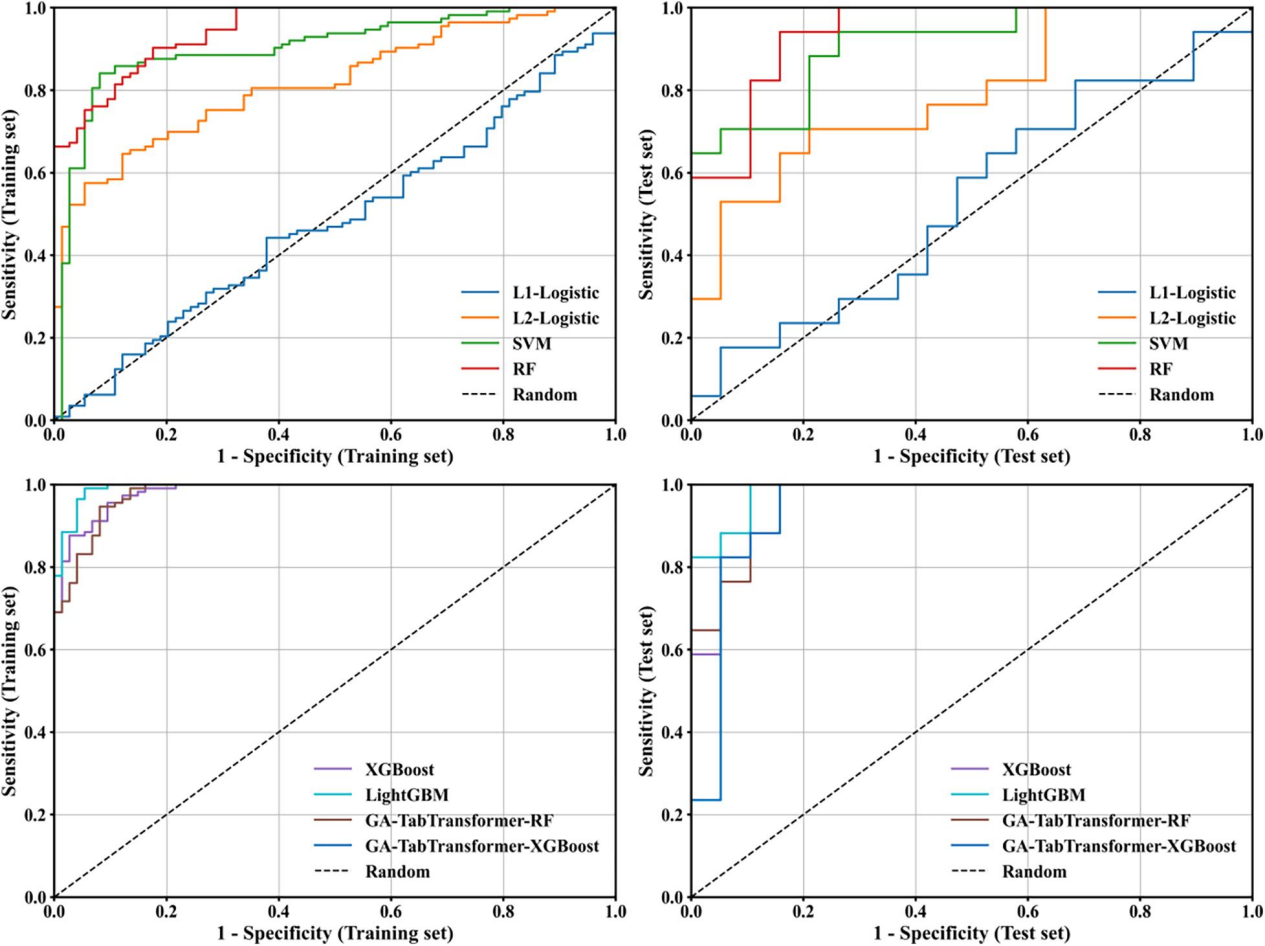
ROC curves of binary classification models.

Model performance was evaluated by nested cross-validation (outer layer 5-fold stratified sampling, inner layer GA parameter tuning), and the main evaluation indicator was the test set AUC value (Table 8). The results showed that the 11 screened variables showed stable predictive efficiency in multiple models. Among them, GA-TabTransformer-XGBoost (AUC = 0.931, 95%CI: 0.857–0.999), LightGBM (AUC=0.908, 95%CI: 0.844–0.971), and XGBoost (AUC = 0.901, 95%CI: 0.833–0.970) all had test set AUC exceeding 0.9, which was significantly better than traditional machine learning models (L1-Logistic AUC = 0.506, SVM AUC = 0.860). Stratified analysis showed that albumin (SHAP value 0.091), other diagnosis score (0.067), and aspartate aminotransferase (0.053) were the core features of model decision-making, which was consistent with the results of SHAP interpretability analysis. This study also compared XGBoost with TRISS score (AUC = 0.786) and RTS score (AUC = 0.762) in clinical efficacy, confirming that the constructed model has significant additional value (all *P* < 0.01), and fully verifying the effectiveness of the previous variable screening strategy (Figure 6).

**Table 8:** Prediction results of binary classification models.

Model	Data set	ACC	PRE	TPR	TNR	F1	AUC (95% CI)
L1-Logistic	Training set	0.500	0.481	0.491	0.508	0.486	0.500 (0.425–0.576)
	Test set	0.475	0.462	0.524	0.431	0.488	0.506 (0.419–0.592)
L2-Logistic	Training set	0.696	0.945	0.389	0.978	0.55	0.818 (0.801–0.835)
	Test set	0.687	0.933	0.386	0.968	0.524	0.812 (0.741–0.883)
SVM	Training set	0.863	0.895	0.811	0.911	0.851	0.891 (0.877–0.906)
	Test set	0.832	0.846	0.802	0.860	0.818	0.860 (0.774–0.946)
RF	Training set	0.846	0.885	0.782	0.906	0.830	0.937 (0.928–0.947)
	Test set	0.804	0.851	0.732	0.872	0.781	0.877 (0.818–0.937)
XGBoost	Training set	0.902	0.906	0.890	0.914	0.897	0.974 (0.966–0.983)
	Test set	0.804	0.819	0.779	0.827	0.793	0.901 (0.833–0.970)
LightGBM	Training set	0.927	0.925	0.924	0.930	0.925	0.987 (0.982–0.992)
	Test set	0.838	0.857	0.814	0.860	0.828	0.908 (0.844–0.971)
GA-TabTransformer-RF	Training set	0.881	0.907	0.840	0.919	0.872	0.972 (0.962–0.982)
	Test set	0.810	0.849	0.744	0.871	0.785	0.900 (0.830–0.969)
GA-TabTransformer-XGBoost	Training set	0.997	0.994	0.999	0.994	0.996	0.998 (0.996–0.999)
	Test set	0.860	0.873	0.838	0.882	0.852	0.931 (0.857–0.999)

#### 3.4.2 Multi-classification prediction modeling

To verify the feature effectiveness and model generalization ability, this study constructed the patient outcome as a three-class variable: cure group, improvement group, and poor prognosis group (sample ratio 108:130:54). The data distribution was balanced (*P* = 0.342), so the original data was used for modeling. Data division and parameter settings were the same as those of the binary classification model (training set: test set = 8:2). For model performance indicators, except for accuracy, the macro-average method was used for calculation, and the results are shown in Table 9.

**Table 9:** Prediction results of multi-classification models.

Model	Data set	ACC	PRE	TPR	TNR	F1	AUC (95% CI)
RF	Training set	0.501	0.399	0.55	0.767	0.431	0.705 (0.676–0.735)
	Test set	0.475	0.379	0.518	0.755	0.406	0.688 (0.593–0.782)
XGBoost	Training set	0.718	0.751	0.632	0.837	0.65	0.887 (0.877–0.898)
	Test set	0.603	0.635	0.518	0.773	0.518	0.748 (0.646–0.851)
LightGBM	Training set	0.763	0.797	0.708	0.863	0.733	0.928 (0.919–0.937)
	Test set	0.594	0.611	0.533	0.772	0.536	0.757 (0.683–0.831)
GA-TabTransformer-XGBoost	Training set	0.915	0.917	0.899	0.953	0.907	0.985 (0.982–0.988)
	Test set	0.594	0.571	0.544	0.773	0.546	0.768 (0.648–0.887)
GA-TabTransformer-RF	Training set	0.684	0.673	0.717	0.834	0.687	0.896 (0.890–0.902)
	Test set	0.594	0.572	0.599	0.787	0.573	0.785 (0.665–0.905)

PRE, TPR, TNR, F1, and AUC are macro-average values of each group.

**Figure 7:**
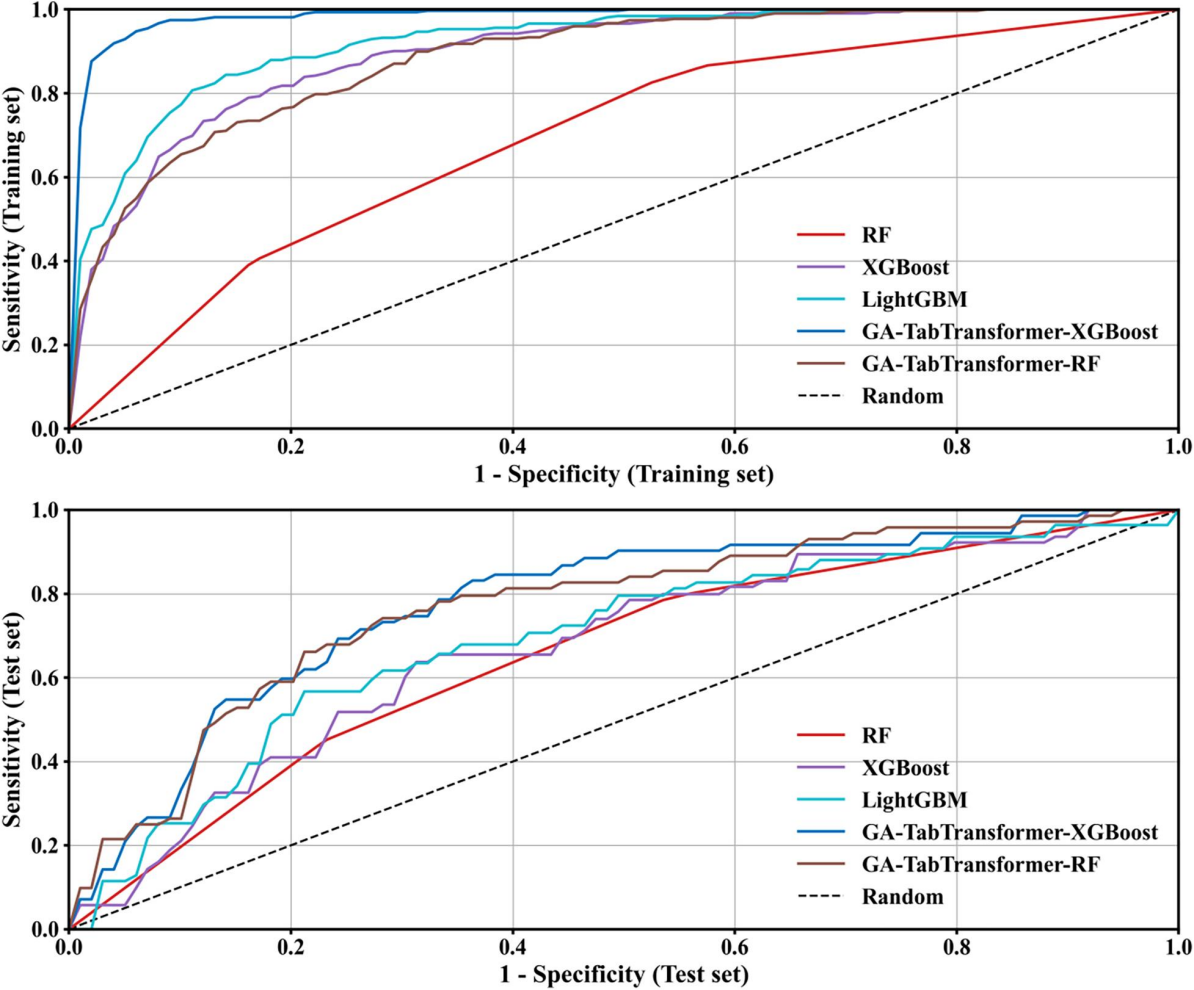
ROC curves of multi-classification models (micro-average strategy).

Multivariate analysis showed that the 11 screened indicators were significantly correlated with the outcome variable (all *P* < 0.05). Model comparison results showed that the GA-TabTransformer-RF model performed the best in the multi-classification task, with a test set macro-average AUC value of 0.785 (95%CI:0.665–0.905) and a macro-average F1 value of 0.573, which was significantly better than traditional models such as RF (0.406) and XGBoost (0.518) (*P* < 0.01). It should be noted that some complex models have the risk of overfitting: the GA-TabTransformer-XGBoost training set AUC reached 0.985, while the test set AUC decreased to 0.768, with a difference of 0.217; the LightGBM training set AUC 0.928 and test set 0.757 had a gap of 0.171, indicating that early stopping strategy and L2 regularization are needed to further optimize the model generalization ability (Figure 7).

**Figure 8:**
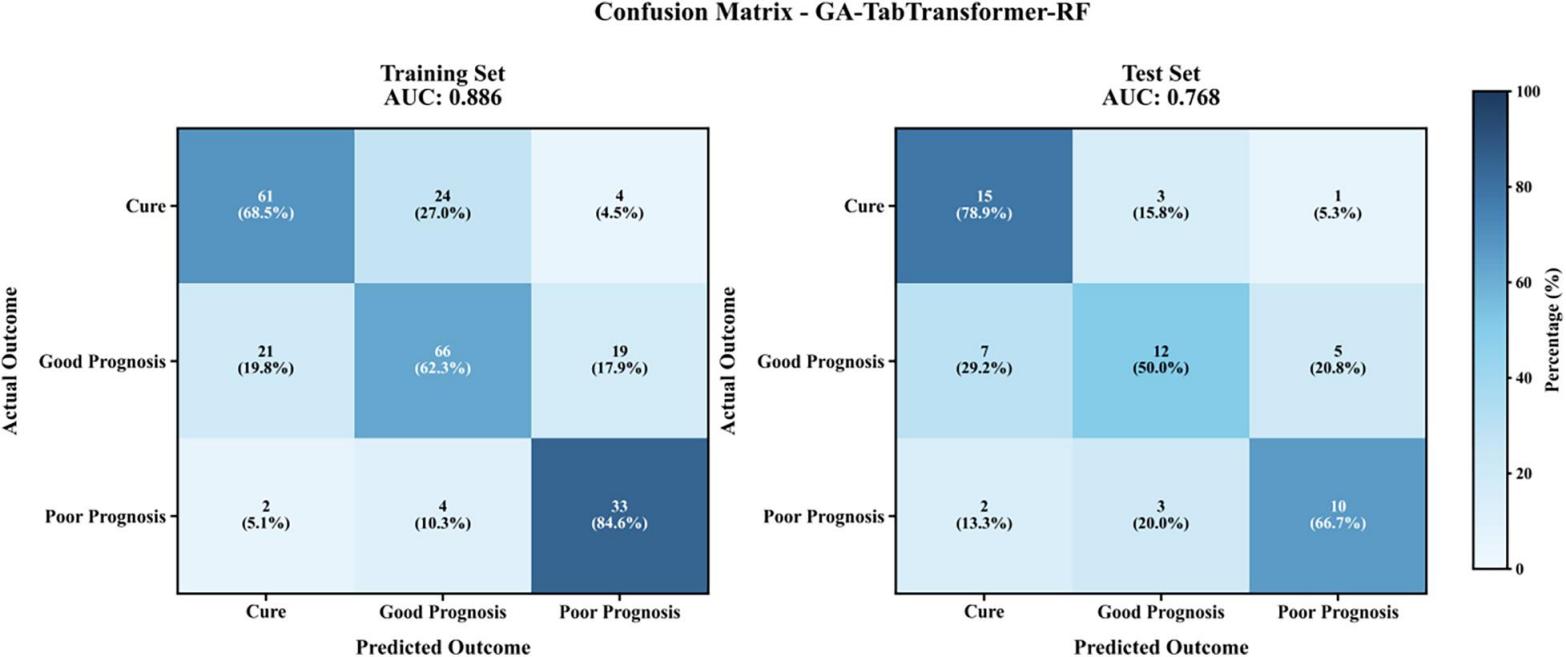
Confusion matrix of the GA-tabTransformer-RF model.

Figure 8 shows the confusion matrix results of the GA-TabTransformer-RF model. The test set macro-average accuracy was 0.594, among which the recognition rate of the poor prognosis group was the highest (TPR = 0.599), followed by the improvement group (TPR = 0.544), and the cure group was the lowest (TPR = 0.533). The specificity of the model for the poor prognosis group reached 0.787, indicating that it has clinical application value in the identification of critically ill patients.

## 4 Discussion

Since the prognostic outcome of severely traumatized patients during hospitalization is affected by many factors, how to accurately evaluate these factors and predict the patient's prognostic outcome has always been an important issue in the field of clinical medicine (24). An effective prediction model can not only provide decision support for clinicians, optimize resource allocation, and improve treatment effects, but also provide personalized treatment plans for patients and reduce unnecessary medical waste (25). This study aims to reveal the main factors affecting patients’ prognostic outcomes by analyzing clinical symptoms, examination results, and medical record text data during hospitalization, and construct an efficient prediction model. This study not only provides a new idea for the prognostic prediction of trauma patients, but also provides a scientific basis for early intervention, risk assessment, and patient management in clinical practice (26).

In this study, text information matching and scoring methods were used to extract important clinical features from patients’ medical records. Through automatic analysis of medical record text, key disease characteristics and clinical manifestations can be effectively identified, avoiding possible subjective bias in the manual extraction process. In addition, the comprehensive utilization of AIS scores of different parts can be realized by adopting objective evaluation methods. The above methods not only improve the efficiency of data processing, but also enhance the utilization value of medical record information. Furthermore, through the feature screening process, 11 variables including main diagnosis score, comprehensive AIS score, albumin, cholinesterase, and creatinine were finally selected as important influencing factors of prognostic outcomes. Among these variables, hemoglobin (Hb), creatinine, etc. overlap with the death-predictive biomarkers found by Prisco et al. (27) in the study of critically ill craniocerebral injury patients in the neurointensive care unit, but this study did not include indicators such as base excess (BE) and mean arterial pressure (MAP). Through the analysis of these variables, the development trend of the patient's condition can be grasped more accurately. In the verification of feature screening and interpretable models, it was found that albumin is a relatively important protective factor, while comprehensive AIS score and diagnosis score are the main risk factors. This finding is consistent with clinical experience and lays a solid theoretical foundation for the subsequent establishment of prediction models.

In the process of constructing the prediction model, we compared a variety of classification algorithms. The main analysis adopted gradient boosting tree (XGBoost) and penalized Logistic regression, and TabTransformer for tabular data was used as sensitivity analysis. The results showed that the GA-TabTransformer-XGBoost model performed the best in the binary classification task (test set AUC = 0.931, 95%CI:0.857–0.999), which was significantly better than traditional models (such as XGBoost with AUC = 0.901 and SVM with AUC = 0.860); the GA-TabTransformer-RF model had a macro-average AUC of 0.785 (95%CI:0.665–0.905) in the multi-classification task, and the recognition rate of the poor prognosis group was the highest (TPR = 0.599, specificity = 0.787). It should be noted that complex models have the risk of overfitting. For example, the AUC difference between the training set and test set of GA-TabTransformer-XGBoost reaches 0.217, indicating that early stopping strategy and L2 regularization are needed to optimize generalization ability.

Through the analysis of clinical data of 292 severely traumatized patients, this study constructed a prognostic prediction model based on 11 core variables. The GA-TabTransformer-XGBoost and GA-TabTransformer-RF models show excellent performance in binary classification and multi-classification tasks, respectively, which can assist clinical optimization of regional trauma treatment strategies: (1) Risk stratification and hierarchical treatment: formulate differentiated disposal lists based on the low/medium/high risk levels output by the model. For example, high-risk patients (predicted probability of poor prognosis >60%) are given priority to start multidisciplinary consultation (neurosurgery, trauma surgery, ICU), medium-risk patients (30%–60%) are arranged for dynamic monitoring and early intervention, and low-risk patients (<30%) can be managed with standardized processes to reduce over-medical treatment. (2) Intelligent resource allocation: dynamically adjust resource priorities according to model prediction results, such as reserving emergency surgical channels for high-risk patients, prioritizing cranial CT plain scan and angiography, and optimizing ICU bed allocation, tilting limited resources to high-risk groups with poor prognosis. (3) Construction of quality control indicator system: Incorporate model prediction accuracy, poor prognosis early warning sensitivity, etc. into regional trauma care quality control indicators, regularly evaluate the treatment efficiency of each hospital, and promote the standardization of treatment processes within the region. Compared with the “clinical + laboratory multi-parameter comprehensive framework” proposed by Li et al., this model focuses on baseline static features at admission, and has higher accessibility and promotion value in primary medical institutions lacking dynamic monitoring data (28). However, it should be acknowledged that the failure to include indicators such as BE, MAP, and PaO2/FiO2 may limit some refinement (27, 28).

## 5 Conclusion

The text matching and scoring method proposed in this study can effectively extract text information from medical records. The 11 core variables screened by the combination of RF and XGBoost show stable predictive efficiency in multiple models. The GA-TabTransformer-XGBoost and GA-TabTransformer-RF models show excellent performance in binary classification and multi-classification tasks, respectively, providing reliable tools for prognostic evaluation of patients in regional trauma care. However, complex models have the risk of overfitting (e.g., the AUC difference between the training set and test set of GA-TabTransformer-XGBoost reaches 0.217). It is recommended to give priority to simple models such as XGBoost in clinical deployment, combined with nomograms to improve interpretability.

## Data Availability

The original contributions presented in the study are publicly available. This data can be found here: https://github.com/ahmulihe/20251104.
