# Supplementary material for: Construction of a predictive early warning model based on machine learning neural network for prognosis of patients with traumatic brain injury
Source: Front Surg. 2026 Mar 9;13:1741425. doi: 10.3389/fsurg.2026.1741425 (PMC13006608; doi:10.3389/fsurg.2026.1741425)
Supplement: Supplementary file 1 [file Supplementaryfile1.docx]

**Appendix A. Supplementary data**

**Table S1 Details on Diagnostic Information**

| Diagnostic Category | Specific Site/Diagnostic Item | Keywords | Score | Objective Function Value |
| --- | --- | --- | --- | --- |
| Main Diagnosis | Head and Craniocerebral | Concussion, craniocerebral injury, skull fracture, cerebral hemorrhage, subdural hematoma, epidural hematoma, cerebral contusion, cerebral edema, increased intracranial pressure, intracranial pressure, cerebral dysfunction, coma, aphasia, hemiplegia, headache, dizziness, nerve injury, cranial CT, MRI examination, electroencephalogram. | 5 | 4.008 |
|  | Neck | Cervical spine injury, cervical fracture, neck soft tissue injury, spinal cord injury, nerve compression, spinal cord lesion, carotid artery injury, neck hemorrhage, nerve root compression, nerve injury, neck X-ray, CT scan, neck MRI, dysphagia. | 1 |  |
|  | Chest | Rib fracture, pulmonary contusion, pneumothorax, hemothorax, cardiac injury, pleural effusion, chest trauma, tracheal injury, airway obstruction, pulmonary infection, respiratory failure, chest CT, chest X-ray, chest ultrasound, impaired lung function. | 2 |  |
|  | Abdomen | Intra-abdominal hemorrhage, splenic rupture, liver injury, intestinal perforation, gastrointestinal injury, bladder injury, peritonitis, abdominal trauma, abdominal CT, abdominal ultrasound, abdominal X-ray, gastrointestinal bleeding, visceral injury. | 3 |  |
|  | Fracture (Extremities/Pelvis) | Fracture, fracture reduction, fracture fixation, extremity fracture, humeral fracture, femoral fracture, tibial fracture, fibular fracture, pelvic fracture, hip fracture, joint dislocation, open fracture, fracture complication, fracture healing, fracture imaging, X-ray, CT scan. | 1 |  |
|  | Spine and Spinal Cord | Spinal fracture, spinal cord injury, spinal dislocation, spondylolisthesis, spinal cord compression, intervertebral disc herniation, spinal deformity, spinal CT, MRI scan, spinal cord dysfunction, paralysis, paraplegia, nerve injury, nerve conduction block. | 3 |  |
|  | Multiple Injuries | Multiple trauma, multi-site injury, systemic injury, trauma syndrome, traumatic shock, sepsis, acute renal failure, multiple organ dysfunction syndrome, severe trauma, systemic infection, trauma assessment, trauma scoring, Injury Severity Score (ISS, TRISS). | 5 |  |
|  | - | - | - |  |
| Other Diagnoses | Hemorrhagic | Bleeding, hematoma, internal bleeding, external bleeding, hemorrhagic shock, vascular rupture, blood loss, acute blood loss, hemostasis, hypotension, decreased hemoglobin, thrombocytopenia, coagulation dysfunction, blood loss volume, plasma transfusion, thrombosis, bleeding assessment, traumatic bleeding, upper gastrointestinal bleeding, lower gastrointestinal bleeding, cerebral hemorrhage, traumatic cerebral hemorrhage, intra-abdominal hemorrhage, traumatic thoracic hemorrhage. | 5 | 42.005 |
|  | Organ Injury | Organ injury, liver injury, kidney injury, splenic rupture, pulmonary contusion, gastrointestinal injury, renal failure, visceral rupture, visceral laceration, hepato-splenic injury, visceral ischemia, visceral dysfunction, liver injury indicators, kidney injury indicators, intra-abdominal visceral injury, traumatic organ injury. | 1 |  |
|  | Cardiovascular | Cardiac injury, pericardial effusion, myocardial injury, cardiac insufficiency, arrhythmia, cardiac arrest, blood pressure fluctuation, hypotension, hypertension, cardiac trauma, arterial injury, vascular rupture, heart failure, pericarditis, traumatic cardiac injury, cardiac resuscitation, arterial embolism, venous thrombosis, coronary artery injury, peripheral vascular injury, electrocardiogram, echocardiogram. | 5 |  |
|  | Soft Tissue | Soft tissue injury, muscle injury, ligament tear, muscle contusion, skin trauma, skin laceration, skin blister, nerve injury, soft tissue swelling, tissue ischemia, wound infection, soft tissue necrosis, soft tissue incision, traumatic nerve injury, deep soft tissue injury, acute soft tissue injury, soft tissue repair. | 1 |  |
|  | Fracture | Fracture, fracture type, open fracture, closed fracture, fracture reduction, fracture healing, fracture complication, extremity fracture, pelvic fracture, femoral fracture, humeral fracture, spinal fracture, fracture fixation, fracture surgery, fracture diagnosis, decreased bone density, fracture imaging, fracture treatment, traumatic fracture, fracture with infection. | 1 |  |
|  | Pyramid (Pyramidal Tract) | Pyramidal tract injury, upper motor neuron injury, pyramidal dysfunction, hemiplegia, limb paralysis, dystonia, loss of deep sensation, pyramidal insufficiency, limb weakness, spinal cord injury, nerve injury, neurological dysfunction, brainstem injury, motor dysfunction, extrapyramidal disorder. | 1 |  |
|  | Underlying Diseases | Diabetes mellitus, hypertension, coronary heart disease, chronic kidney disease, heart disease, liver disease, lung disease, tumor, geriatric disease, immunosuppression, metabolic disease, cardiovascular disease, endocrine disease, digestive system disease, respiratory system disease, renal insufficiency, chronic lung disease, postoperative complication, underlying medical history, chronic disease. | 4 |  |
|  | - | - | - |  |
